# Utilization of Heavily T2-Weighted Imaging and Dynamic Contrast-Enhanced 3T Magnetic Resonance Angiography for Diagnosing Spinal Extradural Arteriovenous Fistula: A Case Report

**DOI:** 10.7759/cureus.76708

**Published:** 2024-12-31

**Authors:** Hayato Murakami, Hironori Mizutani, Keiichi Nakahara, Hiroyuki Uetani, Yasuyuki Kaku, Makoto Nakajima, Akitake Mukasa, Toshinori Hirai, Mitsuharu Ueda

**Affiliations:** 1 Department of Neurology, Graduate School of Medical Sciences, Kumamoto University, Kumamoto, JPN; 2 Department of Diagnostic Radiology, Faculty of Life Sciences, Kumamoto University, Kumamoto, JPN; 3 Department of Neurosurgery, Kumamoto University, Kumamoto, JPN; 4 Department of Neurology, Graduate School of Medical Sciences. Kumamoto University, Kumamoto, JPN

**Keywords:** dynamic contrast-enhanced 3t magnetic resonance angiography, extradural arteriovenous fistula, heavily t2-weighted imaging, long spinal cord lesions, spinal angiography

## Abstract

Spinal arteriovenous fistula (SAVF), including spinal dural arteriovenous fistula and spinal extradural arteriovenous fistula (SEAVF), is a rare spinal vascular disorder characterized by abnormal intradural and extradural arteriovenous shunting, often resulting in progressive motor, sensory, and autonomic dysfunction. Due to its nonspecific presentation and overlapping imaging findings with other spinal diseases, SAVF is frequently misdiagnosed, delaying appropriate treatment and increasing the risk of neurological deterioration. Here, we present a case in which heavily T2-weighted imaging and dynamic contrast-enhanced magnetic resonance angiography at 3T were valuable for the diagnosis of SEAVF.

## Introduction

Spinal arteriovenous fistula (SAVF) is a disease characterized by abnormal connections between spinal arteries and veins, allowing blood to bypass the capillary network and resulting in severe neurological symptoms, such as lower limb numbness, gait disturbance, or bladder-bowel dysfunction [1, 2]. Due to its rarity and limited recognition [3], SAVF is difficult to diagnose and is often initially misinterpreted as another condition [4]. SAVF is classified into two subtypes: spinal dural arteriovenous fistula (SDAVF) and spinal extradural arteriovenous fistula (SEAVF) [5]. Although SDAVF forms a shunt within the spinal veins, SEAVF forms a shunt in the extradural space, creating a shunted venous pouch within the spinal canal or intervertebral foramen [6]. Rapidly progressive spinal disorders associated with long spinal lesions are frequently mistaken for myelitis, leading to immunotherapy treatments such as steroids or high-dose intravenous immunoglobulin (IVIg) [7, 8]. However, immunotherapy is ineffective and may exacerbate neurological symptoms in patients with SAVF [9, 10]. Without timely and accurate diagnosis, SAVF can progress to severe spinal paralysis [11]. This report described a case of SEAVF in which heavily T2-weighted imaging (heavily T2WI) and dynamic contrast-enhanced magnetic resonance angiography at 3T (DCE-MRA) facilitated the detection of abnormal spinal vessels. Heavily T2WI enhances water signals and offers clearer contrast between soft tissues and cerebrospinal fluid, aiding the identification of subtle vascular abnormalities within the spinal canal [12, 13]. DCE-MRA improves image quality with higher magnetic fields while reducing acquisition time [14]. These imaging techniques enabled an accurate diagnosis in a patient exhibiting slowly progressive muscle weakness, sensory disturbance in the lower limbs, and bladder-bowel dysfunction.

## Case presentation

A 61-year-old man with a history of hypertension and type 2 diabetes developed low back pain and left leg pain 7 months before visiting our hospital. The pain gradually spread from the toes to the groin. Five months before the visit, sensory impairment began in the right lower limb, progressing similarly from the toes to the groin. Over time, he experienced bilateral lower limb weakness, more severe on the left side, along with bladder-bowel dysfunction. Two months before admission, he began falling frequently and required a cane for ambulation.

Neurological examination revealed no cognitive impairments or cranial nerve abnormalities. Muscle strength in the neck and upper limbs was intact, whereas lower limb weakness was evident. Bilateral patellar and Achilles tendon reflexes were diminished, and Babinski and Chaddock signs were negative. Romberg’s sign was positive, and abnormal sensations were noted on both soles. Hypesthesia and loss of pain and temperature sensations were observed in both lower limbs, with decreased vibratory sensation around the medial malleoli and impaired proprioception. No signs of cerebellar dysfunction or sacral sparing were observed. Autonomic symptoms included nocturia, urinary incontinence, and loss of bladder-bowel awareness.

**Figure 1:**
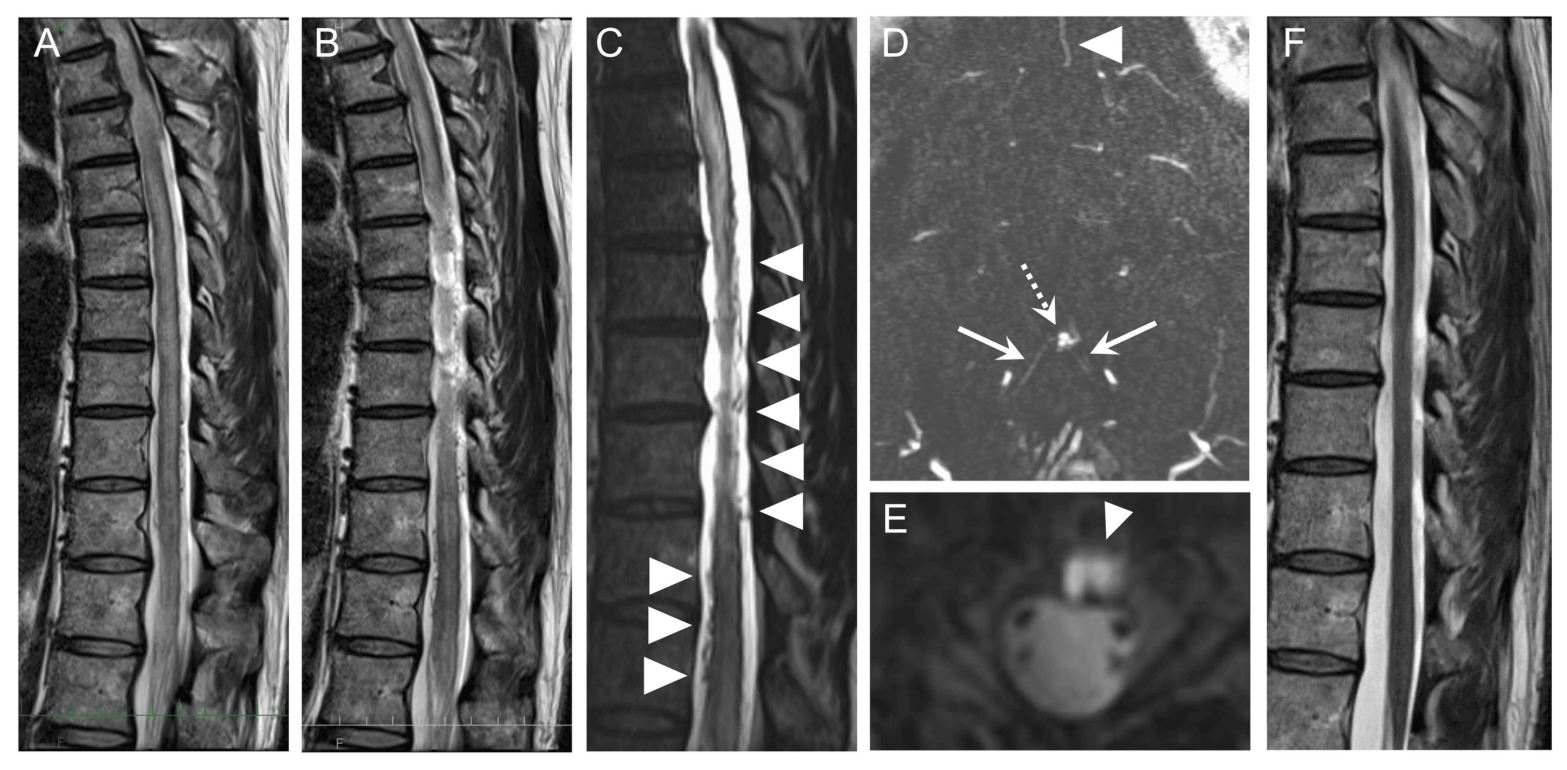
MRI findings in spinal arteriovenous fistula Sagittal T2-weighted magnetic resonance imaging (MRI) of the thoracic and lumbar spine showed diffuse hyperintensity from the upper thoracic cord to the conus, consistent with edema (A, B). Heavily T2-weighted images (heavily T2WI) demonstrated abnormal vascular flow voids in the ventral aspect of the thecal sac, suggesting a dural arteriovenous fistula (arrowheads) (C). Dynamic contrast-enhanced MR angiography (DCE-MRA) showed the shunted pouch (dotted arrow), feeding artery (solid arrows), and draining vein (arrowheads) in the coronal plane (D). A fusion image combining the reconstructed axial DCE-MRA and heavily T2WI demonstrated the presence of a venous pouch within the extradural space (arrowhead) (E). Four months after the treatment, MRI showed resolution of the abnormal signal intensity within the cord (F).

Blood examinations, including a complete blood count and biochemical panels, were unremarkable. Autoimmune markers, such as antinuclear antibodies, anti-SS-A/SS-B antibodies, myeloperoxidase-specific antineutrophil cytoplasmic antibody (MPO-ANCA), proteinase 3-specific antineutrophil cytoplasmic antibody (PR3-ANCA), and anti-aquaporin-4 antibodies, were negative. Vitamin B1, B12, and folate levels were within the normal range. Cerebrospinal fluid (CSF) analysis revealed mild xanthochromia, elevated cell counts (16 cells/µL, 93% of which were mononuclear cells), and increased protein levels (146.8 mg/dL). The IgG index was 0.65, and oligoclonal bands were absent. Contrast-enhanced brain magnetic resonance imaging (MRI) revealed no abnormalities. Spinal MRI showed intramedullary high-intensity signals with swelling extending from the second thoracic level to the conus medullaris. Although flow voids were suspected on T2WI, definitive confirmation was not achieved (Figure 1A, 1B).

**Figure 2:**
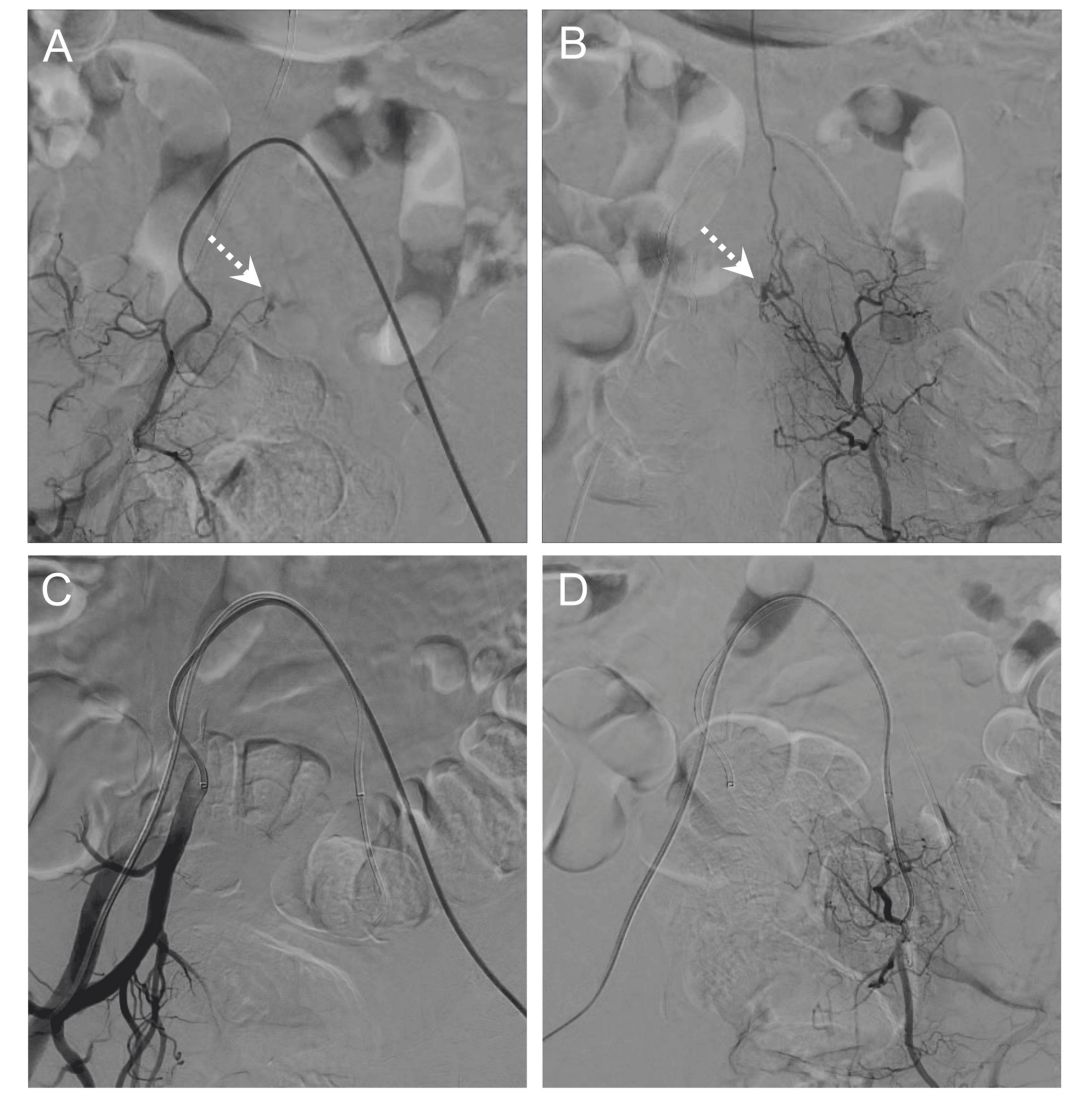
Spinal angiogram Spinal angiography demonstrated the spinal extradural arteriovenous fistula with an arterial feeder from the bilateral S1 segmental arteries branching from the bilateral lateral sacral arteries, forming a venous pouch (dotted arrows) on the posterior surface of the S1 vertebral body (A: right, B: left). Spinal angiography showed stable, complete occlusion of the fistula with no venous shunt flow after embolization (C: right, D: left).

Progressive bilateral lower limb weakness, sensory disturbances, and bladder-bowel dysfunction in an elderly man raised suspicion of SAVF. Additional heavily T2WI revealed dilated and tortuous abnormal vessels (flow voids) extending from the lower thoracic to lumbar levels (Figure 1C). DCE-MRA identified the shunt level, suggesting that the lateral sacral arteries served as feeders (Figure 1D). Furthermore, the reconstructed axial heavily T2WI suggested that the abnormal venous pouch was located within the epidural space (Figure 1E). Spinal angiography identified the bilateral S1 segmental arteries branching from the bilateral lateral sacral arteries as feeders, forming a venous pouch on the posterior surface of the S1 vertebral body. The venous drainage flowed into the perimedullary veins through a bridging vein (Figure 2A, 2B). Following the diagnosis of SEAVF, endovascular embolization was performed, successfully eliminating the shunt (Figure 2C, 2D). Postoperative MRI showed a reduction in abnormal intramedullary signals and resolution of the flow voids (Figure 1F). The patient experienced significant improvements in muscle strength, sensory deficits, and bladder-bowel function after surgery. Sensory disturbances in the lower limbs improved, and urinary incontinence diminished, allowing for the removal of the urethral catheter. Four months after surgery, the patient could walk using only lower limb orthotic devices.

## Discussion

This case illustrates a SEAVF presenting with gradually progressive lower limb weakness, sensory disturbances, and bladder-bowel dysfunction. Heavily T2WI and DCE-MRA played crucial roles in detecting abnormal spinal vessels, leading to a definitive diagnosis.

SEAVF is initially misdiagnosed in over 70% of cases, often posing significant diagnostic challenges [15]. In our case, neuropathy was initially suspected based on clinical findings, and radiological findings on routine MRI suggested probable myelitis. However, male sex, advanced age, lower limb weakness, sensory disturbances, bladder-bowel dysfunction, and the extent of the intraspinal lesion reaching the conus medullaris raised the possibility of SDAVF. The additional heavily T2WI provided decisive evidence by revealing a flow void. This advanced imaging technique likely detected flow voids that conventional T2WI had missed. Oda et al. highlighted the utility of DCE-MRA in localizing SDAVF [14], which was similarly demonstrated in our case by identifying the feeder and fistula point. Reconstructed axial images from heavily T2WI further demonstrated the formation of a venous pouch in the extradural space. Conversely, spinal angiography confirmed the venous pouch, leading to the diagnosis of SEAVF. SEAVF involves an arteriovenous shunt in the extradural space and, in some instances, includes intramedullary shunting, producing symptoms similar to SDAVF. These cases are classified as SEAVF type A. In contrast, cases without intramedullary shunting, where symptoms arise from nerve and spinal cord compression by dilated extradural veins, are classified as type B [6]. The clinical presentation in our case aligns with SEAVF type A, closely resembling SDAVF [16].

Spinal MRI with T2WI is useful for diagnosing SAVF, especially when high-intensity intramedullary signals and flow voids are present [15, 17]. However, distinguishing SAVF from myelitis becomes challenging when only an intramedullary hyperintensity lesion is present. Studies indicate that 58% of SDAVF cases are not correctly diagnosed with the initial spinal MRI [4], and 73% exhibit long spinal lesions resembling those observed in conditions like neuromyelitis optica [18, 19]. Involvement of the conus medullaris may provide a critical diagnostic clue for conditions beyond typical myelitis. The average duration from SEAVF onset to diagnosis is reportedly 11.8 months, during which misdiagnoses such as myelitis or chronic inflammatory demyelinating polyradiculoneuropathy (CIDP)frequently occur [15]. Several reports have documented rapid neurological deterioration following intravenous steroid administration in misdiagnosed cases of SDAVF [15, 20]. This worsening is attributed to the mineralocorticoid effects of steroids, which promote fluid retention and further elevate venous pressure within the lesions [10]. Therefore, prompt and accurate diagnosis of SAVF is essential to prevent unnecessary treatments and mitigate the risk of worsening neurological symptoms.

## Conclusions

This case highlights the importance of considering SAVF in the differential diagnosis of patients with long spinal lesions extending to the conus medullaris, particularly when accompanied by motor, sensory, and autonomic symptoms. Heavily T2WI and DCE-MRA proved useful in detecting abnormal vessels, facilitating early diagnosis and appropriate treatment. Incorporating heavily T2WI and DCE-MRA into diagnostic protocols may enhance the detection of vascular abnormalities in cases with similar clinical presentations.
